# Association between trichomoniasis and prostate and bladder diseases: a population-based case–control study

**DOI:** 10.1038/s41598-022-19561-2

**Published:** 2022-09-13

**Authors:** Hung-Yi Yang, Ruei-Yu Su, Chi-Hsiang Chung, Kuo-Yang Huang, Hsin-An Lin, Jui-Yang Wang, Chien-Chou Chen, Wu-Chien Chien, Hsin-Chung Lin

**Affiliations:** 1 Division of Clinical Pathology, Department of Pathology, Tri-Service General Hospital, National Defense Medical Center, Taipei, Taiwan; 2 School of Public Health, National Defense Medical Center, Taipei, Taiwan; 3 Taiwanese Injury Prevention and Safety Promotion Association, Taipei, Taiwan; 4 Graduate Institute of Pathology and Parasitology, National Defense Medical Center, Taipei, Taiwan; 5 Division of Infection, Department of Medicine, Tri-Service General Hospital SongShan Branch, National Defense Medical Center, Taipei, Taiwan; 6 Department of Family Medicine, Tri-Service General Hospital SongShan Branch, National Defense Medical Center, Taipei, Taiwan; 7 Division of Nephrology, Department of Medicine, Tri-Service General Hospital SongShan Branch, National Defense Medical Center, Taipei, Taiwan; 8 Department of Medical Research, Tri-Service General Hospital, National Defense Medical Center, Taipei, Taiwan; 9 Graduate Institute of Life Sciences, National Defense Medical Center, Taipei, Taiwan

**Keywords:** Microbiology, Oncology, Pathogenesis, Risk factors, Urology

## Abstract

*Trichomonas vaginalis* infection is one of the most widespread sexually transmitted infections in the world. There are approximately 276 million cases worldwide. Most men remain undiagnosed and untreated because they are asymptomatic. The chronic inflammation induced by persistent infection may increase the risk of developing genitourinary cancers. In this study, we aimed to investigate the association between trichomoniasis and benign prostate hyperplasia (BPH), prostate cancer (PCa), and bladder cancer (BC) in Taiwan. We designed a case–control study by using the database of the National Health Insurance program in Taiwan. We used the International Classification of Diseases, 9th Revision classifications to classify all the medical conditions in the case and control groups. All odds ratios (ORs) and 95% confidence intervals (CIs) were analyzed using multivariable logistic regression to adjust for all comorbidities and variables. From 2000 to 2015, we enrolled a total of 62,544 individuals as the case group and 187,632 as the control group. Trichomoniasis exposure had a significant association with BPH and PCa (adjusted OR: BPH = 2.685, 95% CI = 1.233–4.286, *P* = 0.013; PCa = 5.801, 95% CI = 1.296–26.035, *P* = 0.016). The relative risk was much higher if patients had both trichomoniasis and depression (adjusted OR = 7.682, 95% CI = 5.730–9.451, *P* < 0.001). Men with trichomoniasis had a significantly higher risk of developing BPH and PCa than those without. Healthcare professionals should not only pay more attention to disease treatment, but also to public health education.

## Introduction

Benign prostate hyperplasia (BPH), prostate cancer (PCa), and bladder cancer (BC) are common diseases in the elderly male population. The pathological mechanism of these diseases is not yet fully understood. Inflammation of the prostate, which can cause proliferation of epithelium and stroma, is considered to be related to both BPH and PCa^1,2^. In addition, urinary tract infection (UTI) is significantly associated with genitourinary cancers (GUC), including kidney, prostate, and bladder cancers^3^. *Trichomonas vaginalis* infection is one of the most common sexually transmitted infections (STIs), accounting for approximately 276.4 million new cases annually^4^. Because most male patients are asymptomatic and remain undiagnosed and untreated, persistent infection may cause chronic inflammation, which may increase the risk of GUC. There is a lack of research into the relationship between *T. vaginalis* infection and BC; however, some studies have mentioned that *T. vaginalis* infection may induce proliferation of prostatic epithelial cells and stromal cells^5,6^. Some in vitro studies showed that PCa may be associated with the up-regulation of the expression of genes that can control cell apoptosis or be overexpressed as a proto-oncogene^7,8^. The study from Vienna General Hospital discovered that 29/86 (33.7%) patients with BPH were positive for *T. vaginalis* on polymerase chain reaction (PCR) testing^9^. The Health Professionals Follow-up Study (HPFS) demonstrated that *T. vaginalis* seropositivity had a positive correlation with PCa risk^10^. However, conflicting results have also been reported. Miguelle et al. demonstrated that there was no significant association between *T. vaginalis* infection and PCa in Caucasian or African-American groups^11^. Another multicenter study in the USA revealed that patients with a history of STIs and positive STI serologies demonstrated no association with BPH^12^. In addition, there is still a lack of related literature regarding BC and Asian male populations. Thus, this study aimed to examine the association between *T. vaginalis* infection and BPH, BC, or PCa.

## Materials and methods

### Data source

We designed a population-based nationwide nested case–control study and obtained inpatient and outpatient files from Taiwan’s National Health Insurance Research Database (NHIRD). The data were collected from the Longitudinal Health Insurance Database 2005 (LHID2005), a part of NHIRD. We randomly selected approximately 2,000,000 people among the total population. All personal information was encrypted by National Health Research Institutes before released.

### Ethical approval

Our study was approved by the Institutional Review Board of Tri-Service General Hospital, National Defense Medical Center, Taipei, Taiwan (TSGHIRB No.: B-109-31). All stages of the study were carried out in accordance with relevant guidelines and regulations. Because the patient identifiers were encrypted before their data were used for research purposes to protect confidentiality, the requirement for written informed consent from patients for data linkage was waived by Institutional Review Board of Tri-Service General Hospital, National Defense Medical Center, Taipei.

### Identification of the case and control groups

**Figure 1:**
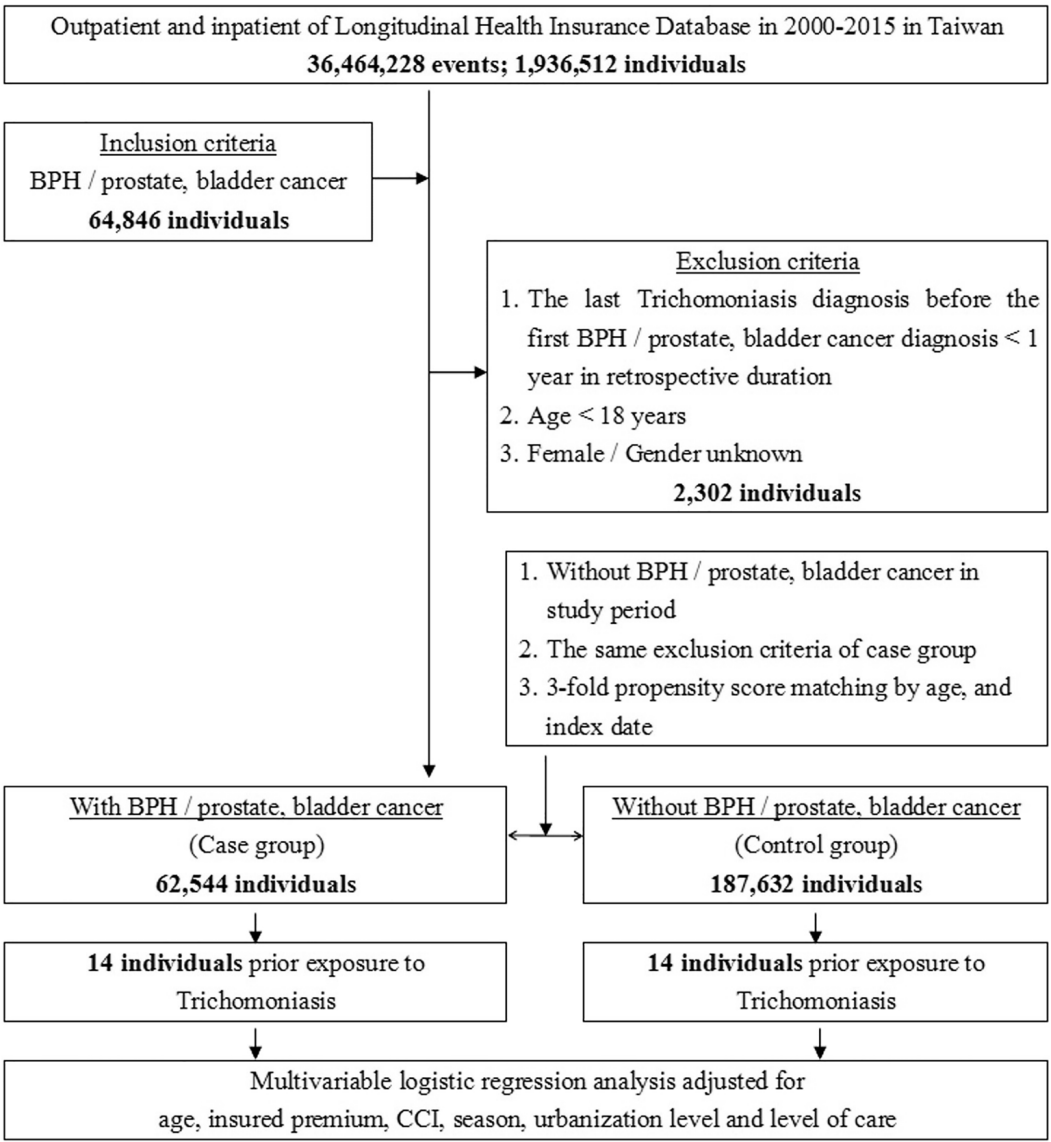
The flowchart of the study design (nested case–control study) from National Health Insurance Research Database in Taiwan.

We selected patients from 2000 to 2015 who had been diagnosed with BPH, PCa, or BC based on the International Classification of Diseases, 9th Revision, Clinical Modification (ICD-9-CM) codes as the case group (Table S1). We defined the date of the first disease diagnosis as the index date. We also used ICD-9-CM codes to identify patients with *T. vaginalis* infection (Table S1). In contrast, the control groups were patients without BPH, PCa, or BC. Among all patients in the case and control groups, we not only selected patients in a 1:3 case:control ratio, matching based on age and index date, but also excluded (1) women and patients of unknown sex, (2) patient’s aged less than 18 years, and (3) those last diagnosed with trichomoniasis within 1 year before the index date (Fig. 1). The matching method was taken propensity score matching, wherein match tolerance was set at 0.15. The propensity score matching was set as using logistic regression in estimation algorithm and nearest neighbor matching in matching algorithm. The options for nearest neighbor were random in matching order, non-replacement, 1 to 3 matching, and no caliper. The comorbidities in our study included hypertension, myocardial infarction, congestive heart failure, cerebral or peripheral vascular disease, dementia, chronic obstructive pulmonary disease (COPD), type 2 diabetes, renal disease, and malignant disease except PCa and BC. We also evaluated depression as one of the comorbidities in our study because it may be associated with some cancers^13^.

### Covariates for analysis

The covariates in our study included age group (18–44, 45–64, ≥ 65 years), four seasons (spring, summer, autumn and winter), with or without diagnosis of depression, geographical area of residence (north, center, south, east and outlying islands of Taiwan), urbanization level of residence (levels 1 to 4), levels of hospitals as medical centers, regional and local hospitals, and monthly income (in New Taiwan Dollars [NT$]; < 18,000, 18,000–34,999, ≥ 35,000). The urbanization level of residence was defined according to the population, along with various indicators of the level of political, economic, cultural, and metropolitan development. Level 1 was defined as a population of > 1,250,000, and a specific designation as political, economic, cultural, and metropolitan development. Level 2 was defined as a population between 500,000 and 1,249,999, and as playing an important role in the political system, economy, and culture. Urbanization levels 3 and 4 were defined as a population between 149,999 and 499,999, and < 149,999, respectively.

### Statistical analysis

The statistical analyses were performed using SPSS version 22.0 (IBM Corp, Armonk, NY, USA). A P-value < 0.05 was considered significant. The Chi-squared or Fisher exact test was used to evaluate distributions between the case and control groups. Continuous variables were evaluated using the *t* test. Unconditional multiple logistic regression analyses were performed to evaluate the risks of BPH, PCa, and BC associated with trichomoniasis after adjusting for age, insurance premium, comorbidities, season, urbanization, and level of care. Adjusted models with significant covariates were constructed using background selection with the likelihood ratio test.

## Results

### Demographic characteristics of the study population

Table 1 demonstrates the population distribution of different characteristics for 62,544 cases and 187,632 controls from 2000 to 2015. There were no significant differences in age between groups after matching. The proportion with trichomoniasis in the case group was 0.02% (14/62,544), while it was 0.01% (14/187,632) in the control group (*P* < 0.001).

**Table 1:** Characteristics of the study group.

BPH/prostate cancer, bladder cancer Variables	Total		With		Without		*P*
	n	%	n	%	n	%	
**Total**	250,176		62,544	25.00	187,632	75.00	
**Trichomoniasis**							0.004
Without	250,148	99.99	62,530	99.98	187,618	99.99	
With	28	0.01	14	0.02	14	0.01	
**Age (years)**	73.15 ± 11.41		73.21 ± 10.65		73.13 ± 11.65		0.129
**Age group (years)**							0.999
18–44	2664	1.06	666	1.06	1998	1.06	
45–64	50,292	20.10	12,573	20.10	37,719	20.10	
≥ 65	197,220	78.83	49,305	78.83	147,915	78.83	
**Insurance premium (NT$)**							< 0.001
< 18,000	245,698	98.21	61,654	98.58	184,044	98.09	
18,000–34,999	3654	1.46	712	1.14	2942	1.57	
≥ 35,000	824	0.33	178	0.28	646	0.34	
**Depression**							< 0.001
Without	217,896	87.10	50,509	80.76	167,387	89.21	
With	32,280	12.90	12,035	19.24	20,245	10.79	
**CCI_R**	1.74 ± 2.96		1.71 ± 2.77		1.75 ± 3.03		< 0.001
**Season**							< 0.001
Spring (Mar–May)	56,893	22.74	15,495	24.77	41,398	22.06	
Summer (Jun–Aug)	60,567	24.21	15,709	25.12	44,858	23.91	
Autumn (Sep–Nov)	72,621	29.03	16,666	26.65	55,955	29.82	
Winter (Dec–Feb)	60,095	24.02	14,674	23.46	45,421	24.21	
**Location**							< 0.001
Northern Taiwan	99,711	39.86	26,475	42.33	73,236	39.03	
Central Taiwan	71,555	28.60	16,878	26.99	54,677	29.14	
Southern Taiwan	63,601	25.42	14,985	23.96	48,616	25.91	
Eastern Taiwan	14,366	5.74	3957	6.33	10,409	5.55	
Outlying islands	943	0.38	249	0.40	694	0.37	
**Urbanization level**							< 0.001
1 (Highest)	75,256	30.08	18,936	30.28	56,320	30.02	
2	113,122	45.22	29,293	46.84	83,829	44.68	
3	17,865	7.14	4119	6.59	13,746	7.33	
4 (Lowest)	43,933	17.56	10,196	16.30	33,737	17.98	
**Level of care**							< 0.001
Hospital center	89,122	35.62	23,060	36.87	66,062	35.21	
Regional hospital	115,596	46.21	26,602	42.53	88,994	47.43	
Local hospital	45,458	18.17	12,882	20.60	32,576	17.36	

*P:* Chi-square/Fisher exact test on categorical variables and *t* test on continue variables.

### Variable evaluation in the multiple logistic regression

We present the results of the multivariable logistic regression analyses in Table 2. Patients with trichomoniasis had a significantly higher risk of BPH, PCa, or BC (adjusted odds ratio [AOR] = 2.999, 95% confidence interval [CI] = 1.426–5.301, P = 0.002). There was also a significantly higher risk for patients with depression (AOR = 3.124, 95% CI = 1.808–4.838, *P* < 0.001). The opposite result was noted in patients with middle or high insurance premiums (insurance premium NT$18,000–34,999: AOR = 0.745, 95% CI = 0.688–0.799, *P* < 0.001; insurance premium > NT$35,000: AOR = 0.836, 95% CI = 0.701–0.979, *P* = 0.019). Patients diagnosed in summer, autumn, or winter also had significantly lower risk than the control group (summer: AOR = 0.938, 95% CI = 0.902–0.953, *P* < 0.001; autumn: AOR = 0.790, 95% CI = 0.758–0.805, *P* < 0.001; winter: AOR = 0.862, 95% CI = 0.824–0.878, *P* < 0.001). Patients who lived in areas with a higher urbanization level had a significantly higher risk of BPH, PCa, or BC (urbanization level 1: AOR = 1.160, 95% CI = 1.124–1.189, *P* < 0.001; urbanization level 2: AOR = 1.211, 95% CI = 1.179–1.235, *P* < 0.001) but had significantly lower risk when diagnosed at a higher level of care (hospital center: AOR = 0.819, 95% CI = 0.796–0.902, *P* < 0.001; regional hospital: AOR = 0.745, 95% CI = 0.724–0.808, *P* < 0.001) instead.

**Table 2:** Risk of BPH/prostate cancer and bladder cancer based on stated variables analyzed using multivariable logistic regression.

Variables	Crude OR	95% CI	95% CI	*P*	Adjusted OR	95% CI	95% CI	*P*
**Trichomoniasis**								
Without	Reference				Reference			
With	3.000	1.430	6.294	0.004	2.999	1.426	5.301	0.002
**Age group (years)**								
18–44	Reference				Reference			
45–64	1.000	0.914	1.094	0.999	1.015	0.923	1.107	0.782
≥ 65	1.000	0.915	1.092	0.999	1.006	0.919	1.098	0.794
**Insured premium (NT$)**								
< 18,000	Reference				Reference			
18,000–34,999	0.722	0.665	0.784	< 0.001	0.745	0.688	0.799	< 0.001
≥ 35,000	0.823	0.697	0.971	0.021	0.836	0.701	0.979	0.019
**Depression**								
Without	Reference				Reference			
With	3.286	1.846	4.959	< 0.001	3.124	1.808	4.838	< 0.001
**CCI_R**	0.996	0.993	0.999	0.006	1.000	0.998	1.005	0.058
**Season**								
Spring	Reference				Reference			
Summer	0.936	0.912	0.960	< 0.001	0.938	0.902	0.953	< 0.001
Autumn	0.796	0.776	0.816	< 0.001	0.790	0.758	0.805	< 0.001
Winter	0.863	0.841	0.886	< 0.001	0.862	0.824	0.878	< 0.001
**Location**					Had multicollinearity with urbanization level			
Northern Taiwan	Reference				Had multicollinearity with urbanization level			
Central Taiwan	0.854	0.835	0.873	< 0.001	Had multicollinearity with urbanization level			
Southern Taiwan	0.853	0.833	0.873	< 0.001	Had multicollinearity with urbanization level			
Eastern Taiwan	1.052	1.011	1.094	0.012	Had multicollinearity with urbanization level			
Outlying islands	0.992	0.858	1.148	0.919	Had multicollinearity with urbanization level			
**Urbanization level**								
1 (Highest)	1.113	1.082	1.144	< 0.001	1.160	1.124	1.189	< 0.001
2	1.156	1.127	1.186	< 0.001	1.211	1.179	1.235	< 0.001
3	0.991	0.951	1.033	0.685	0.987	0.952	1.036	0.924
4 (Lowest)	Reference				Reference			
**Level of care**								
Hospital center	0.883	0.861	0.905	< 0.001	0.819	0.796	0.902	< 0.001
Regional hospital	0.756	0.738	0.775	< 0.001	0.745	0.724	0.808	< 0.001
Local hospital	Reference				Reference			

*OR* odds ratio, *CI* confidence interval, *Adjusted OR* adjusted for variables listed in the table.

### Risk of BPH/PCa and BC in the trichomoniasis group stratified by covariates

The risk of BPH, PCa, or BC stratified based on variables using multivariable logistic regression is shown in Table 3. Patients with trichomoniasis had a 2.999 times higher risk of BPH, PCa, or BC than the control group (AOR = 2.999, 95% CI = 1.426–5.301). In the case of trichomoniasis, there were significantly higher risks of BPH, PCa, or BC in patients aged > 65 years old, with lower insurance premiums, with/without depression, first diagnosed in winter, urbanization level 2, and first diagnosed in a local hospital (age > 65 years: AOR = 3.685, 95% CI = 1.704–8.015; insurance premium < NT$18,000: AOR = 2.999, 95% CI = 1.326–5.301; with depression: AOR = 3.104, 95% CI = 1.706–5.972; without depression: AOR = 2.545, 95% CI = 1.138–4.289; first diagnosed in winter: AOR = 4.806, 95% CI = 1.104–19.675; urbanization level 2: AOR = 3.284, 95% CI = 1.057–10.978; first diagnosed in local hospital: AOR = 15.121, 95% CI = 1.762–118.976).

**Table 3:** Risk of BPH/prostate cancer and bladder cancer stratified by variables listed in the table by using multivariable logistic regression.

BPH/prostate, bladder cancer Stratified	With			Without			With vs. Without (reference)			
	Trichomoniasis exposure	Population	%	Trichomoniasis exposure	Population	%	Adjusted OR	95%CI	95%CI	*P*
**Total**	14	62,544	0.022	14	187,632	0.007	2.999	1.426	5.301	0.002
**Age group (years)**										
18–44	0	666	0.000	0	1998	0.000	–	–	–	–
45–64	0	12,573	0.000	2	37,719	0.005	0.000	–	–	0.999
≥ 65	14	49,305	0.028	12	147,915	0.008	3.685	1.704	8.015	0.001
**Insurance premium (NT$)**										
< 18,000	14	61,654	0.023	14	184,044	0.008	2.999	1.426	5.301	0.002
18,000–34,999	0	712	0.000	0	2942	0.000	–	–	–	–
≥ 35,000	0	178	0.000	0	646	0.000	–	–	–	–
**Depression**										
Without	4	50,509	0.008	7	167,387	0.004	2.545	1.138	4.289	< 0.001
With	10	12,035	0.083	7	20,245	0.035	3.104	1.706	5.972	< 0.001
**Season**										
Spring	3	15,495	0.019	1	41,398	0.002	7.745	0.671	70.986	0.175
Summer	2	15,709	0.013	4	44,858	0.009	1.301	0.104	5.258	0.603
Autumn	4	16,666	0.024	6	55,955	0.011	2.197	0.482	4.894	0.224
Winter	5	14,674	0.034	3	45,421	0.007	4.806	1.104	19.675	0.033
**Urbanization level**										
1 (Highest)	2	18,936	0.011	2	56,320	0.004	3.199	0.453	22.845	0.241
2	6	29,293	0.020	6	83,829	0.007	3.284	1.057	10.978	0.035
3	1	4119	0.024	1	13,746	0.007	3.351	0.210	53.777	0.382
4 (Lowest)	5	10,196	0.049	5	33,737	0.015	3.086	0.898	10.801	0.077
**Level of care**										
Hospital center	1	23,060	0.004	3	66,062	0.005	0.965	0.094	9.301	0.886
Regional hospital	7	26,602	0.026	10	88,994	0.011	2.301	0.846	6.127	0.071
Local hospital	6	12,882	0.047	1	32,576	0.003	15.121	1.762	118.976	0.008

*Adjusted OR* adjusted odds ratio: adjusted for the variables listed in Table 2, *CI* confidence interval.

### Risk of BPH/PCa and BC in subgroup with *T. vaginalis* exposure and the joint effect

**Figure 2:**
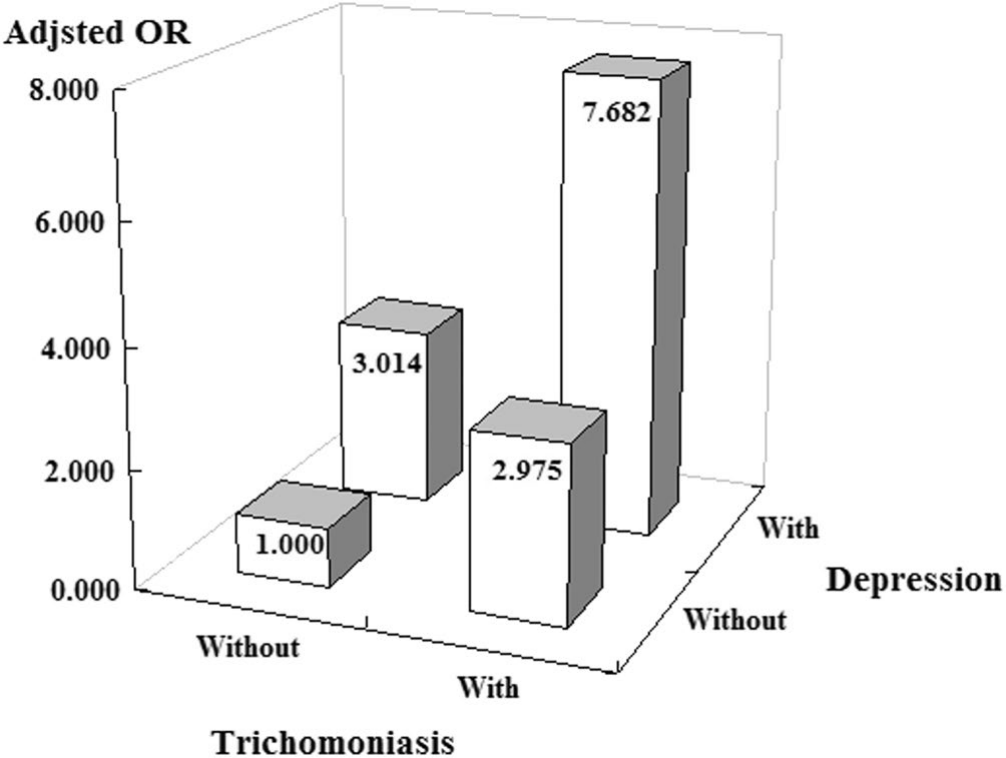
Risk of BPH/prostate or bladder cancer stratified by trichomoniasis and depression status using logistic regression.

Table 4 presents the *T. vaginalis* exposure ratio in each subgroup of BPH/PCa and BC. *T. vaginalis* exposure is significantly associated with a higher risk of BPH and PCa (BPH: AOR = 2.685, 95% CI = 1.233–4.286, *P* = 0.013; PCa: AOR = 5.801, 95% CI = 1.296–26.035, *P* = 0.016), but has no significant association with BC (AOR = 4.012, 95% CI = 0.524–31.145, *P* = 0.151). In addition, patients with both depression and *T. vaginalis* exposure had a significantly higher risk of developing BPH, PCa, or BC in comparison with other groups with only one condition or without them (AOR = 7.682, 95% CI = 5.730–9.451, *P* < 0.001) (Table 5, Fig. 2).

**Table 4:** BPH/prostate cancer and bladder cancer subgroups analyzed using multivariable logistic regression.

BPH/prostate cancer, bladder cancer subgroup	Trichomoniasis exposure	Population	%	Adjusted OR	95%CI	95%CI	*P*
Without	14	187,632	0.007	Reference			
**With**	14	62,544	0.022	2.999	1.426	5.301	0.002
BPH/prostate cancer	13	59,325	0.022	2.995	1.422	4.389	0.003
BPH	11	51,482	0.021	2.685	1.233	4.286	0.013
Prostate cancer	2	6254	0.032	5.801	1.296	26.035	0.016
Bladder cancer	1	3873	0.026	4.012	0.524	31.145	0.151

*Adjusted OR* adjusted odds ratio (adjusted for the variables listed in Table 2), *CI* confidence interval.

**Table 5:** Risk of BPH/prostate cancer or bladder cancer stratified by trichomoniasis and depression status using logistic regression.

Trichomoniasis	Depression	n	Adjusted OR	95% CI	95% CI	*P*
Without	Without	167,387	Reference			
With	Without	50,509	2.975	1.429	3.608	< 0.001
Without	With	20,245	3.014	1.586	4.297	< 0.001
With	With	12,035	7.682	5.730	9.451	< 0.001

*Adjusted OR* adjusted odds ratio (adjusted for variables listed in Table 2), *CI* confidence interval.

## Discussion

We designed this case–control study based on nationwide data from Taiwan NHIRD. We found that *T. vaginalis* infection was significantly associated with BPH and PCa in a male population. Therefore, *T. vaginalis* could be a pathogen that induces BPH and PCa. However, there was no significant association between trichomoniasis and BC. Furthermore, patients with both trichomoniasis and depression had 7.682 times higher risk of developing BPH, PCa, or BC. This result suggests that the joint effect of trichomoniasis and depression could increase the risk of BPH, PCa, or BC.

The mechanism of *T. vaginalis* inducing BPH and PCa still remains unclear. Several studies have demonstrated different possible mechanisms. In women, *T. vaginalis* induces pro-inflammatory cytokine production, including interleukin-6 (IL-6), interleukin-8 (IL-8), and chemokine ligand 2 (CCL2), while attaching to vaginal epithelial cells^14^. A similar inflammatory reaction was also noted in *T. vaginalis*-infected prostatic epithelial cells in some in vitro studies^5,6^. Repeated cell damage and repair in chronic inflammation is likely to play an important role in inducing BPH^15^. Furthermore, the alteration in cytokine expression during chronic inflammation may have effects on cell growth and proliferation of the prostate epithelium and stroma in BPH^15^. The activated mast cells stimulated by *T. vaginalis*-infected prostatic epithelial cells can initiate IL-8 and CCL2 expression^5^. IL-8 could be a predictive marker for BPH^16^. Some in vitro studies demonstrated that IL-8 can stimulate fibroblast growth factor 2 (FGF-2), which causes the mitosis of prostate stromal cells^17^. IL-8 could also cause cyclin D1 expression to promote stromal cells proliferation^18^. In addition, CCL2, secreted by the prostatic stroma fibroblast, could promote both BPH and PCa progression^5^.

*T. vaginalis* possibly induces carcinogenesis of the prostate. The infected prostatic epithelial cells produce IL-6 in chronic inflammation^19^. In early studies, an elevated serum IL-6 level was noted in patients with advanced PCa^20^. The positive correlation between IL-6 receptor expression and cell proliferation has been reported^21^. IL-6 also induces epithelial–mesenchymal transition (EMT) in breast cancer growth and metastasis^22^, and the same reaction may also occur in prostatic epithelial cells^23^. In addition, more than one study has demonstrated that IL-6 could enhance androgen receptor (AR) activity and AR gene expression^24^, which is also related to prostate cancer growth. Twu et al. demonstrated that *T. vaginalis* macrophage migration inhibitory factor (TvMIF) plays an important role in inducing PCa^7^. There are already studies that have proven that higher human macrophage migration inhibitory factor (HuMIF) levels are present in several cancers, including PCa^25^. The structure of TvMIF is similar to that of HuMIF, which might explain why TvMIF also has the ability to promote cell proliferation, sustain inflammation, and stimulate the growth of prostate cancer cells^7^.

In previous studies, *T. vaginalis* could play an important role as a carcinogen of female cervical cancer^26,27^. However, there is no consensus regarding the relationship between trichomoniasis and cervical cancer^28^. Likewise, the role of *T. vaginalis* in the development of PCa is still controversial. Zhu et al. demonstrated that there was a negative association between PCa and trichomoniasis^29^. Instead, they discovered culture supernatant of *T. vaginalis* not only inhibited growth but also induced apoptosis of prostate cancer cell. *T. vaginalis* could enhance anti-proliferative molecules and decrease the expression of anti-apoptotic molecule^29^. The *T. vaginalis* adhesion protein could induce T helper 2 cell cytokines reaction to stimulate the productions of specific antibody^30^. This enhancement of the immune response might suppress the cancer cell activity^31^. Moreover, another further study also showed that *T. vaginalis* seropositivity does not raise mortality risk in men with PCa^32^. The inflammatory response caused by *T. vaginalis* might not have influence in the development and progression of PCa^32^. However, the detail mechanism of immune response between *T. vaginalis* and prostate epithelial cell still remained unclear, further investigations are necessary.

There were still a lack of studies to prove that trichomoniasis is associated with BC. We still included BC patients in our study because the inflammatory cytokines found in trichomoniasis, including IL-6 and IL-8, are also associated with a higher risk of developing BC^33,34^ and some parasites, such as *Schistosoma haematobium*, can induce BC. However, our study shows no significant association between *T. vaginalis* infection and BC probably because of limited sample.

We added depression as one of the comorbidities in our study due to another previous nationwide population-based cohort study in Taiwan which showed that patients with trichomoniasis had higher risks for developing an individual psychiatric disorder, including depression, anxiety, bipolar disorder, schizophrenia and substance abuse^35^. Our study results demonstrate that except for depression, no comorbidities had a significant association with BPH, PCa, or BC. The joint effect of trichomoniasis and depression increased the risk by 7.682 times that of the control group. A recent study showed that depression is associated with decreased immunity^36^. Moreover, depression can also cause cytokine dysregulation and increased serum IL-6 concentration^36^, which might enhance carcinogenesis after *T. vaginalis* infection.

Although this study was a large-scale population-based nationwide design with long-term monitoring from 2000 to 2015, there are still several limitations. First, the NHIRD does not contain detailed information regarding the symptom severity of BPH, the histological and TNM classification of PCa and BC, serum sex hormone concentrations, Prostate-Specific Antigen (PSA) levels, *T. vaginalis* antibody test, family history, or personal history such as sexual exposure, physical activity, alcohol consumption or tobacco smoking. Second, we did not include body mass index (BMI) as one of our variables. Obesity is one of the risk factors for BPH and PCa^37^, which might affect their association with trichomoniasis. Third, our study might underestimate the exact number of patients with trichomoniasis. Most male patients would not seek treatment due to being asymptomatic, and ineffective screening protocols because of the lack of public health awareness could also lead to possible *T. vaginalis* infection being neglected^38^. Another reason that caused underestimation of our case group is that the antibody tests of *T. vaginalis* were not performed popularly during diagnosis and mostly were female patients^39^. It is possible that *T. vaginalis* was substantially undercoded and underrepresented in the study population. Fourth, the number of cases of BC might be too small to be significant and the tracking time might not be sufficient for disease monitoring. Trichomoniasis can be a chronic infection. The outcomes in the study might present later in life, so in some men trichomonas exposure may happen a few years before these outcomes appear or many decades prior to diagnosis. Fifth, the outcome of each case was defined as the first code for BPH, PCa, or BC. This assumes that there is a common pathway between trichomoniasis and these 3 separate diseases. However, this approach method could also ignore these outcomes as comorbidities. For example, patients with PCa or BC could also have BPH or other urinary symptoms. It is possible that many PCa or BC outcomes were ignored if BPH was coded first. This might be another reason that our study samples were underestimated. Sixth, our study was designed as an observational case–control study, so the causation cannot be detected. We hope that in the future more research will support our thesis.

## Conclusion

Male patients with *T. vaginalis* infection have an increased risk of developing BPH and PCa, especially in trichomoniasis patients with comorbid depression. Due to the lack of awareness of this pathogen, clinicians should not only treat patients who are already diagnosed but should also pay more attention to groups with higher trichomoniasis exposure risk.

## Supplementary Information

Supplementary Table S1.

## Data Availability

Data supporting the conclusions of this article are included within the article and its additional files. The datasets used and/or analyzed during the present study will be made available by the corresponding author upon reasonable request.

## Abbreviations

AOR: Adjusted odds ratio
AR: Androgen receptor
BC: Bladder cancer
BMI: Body mass index
BPH: Benign prostate hyperplasia
CCL2: Chemokine ligand 2
CI: Confidence interval
COPD: Chronic obstructive pulmonary disease
EMT: Epithelial–mesenchymal transition
FGF-2: Fibroblast growth factor 2
GUC: Genitourinary cancers
HPFS: Health Professionals Follow-up Study
HuMIF: Human macrophage migration inhibitory factor
IL: Interleukin
LHID2005: Longitudinal Health Insurance Database 2005
NHI: National Health Insurance
NHIRD: National Health Insurance Research Database
NT$: New Taiwan Dollars
OR: Odds ratio
PCa: Prostate cancer
PSA: Prostate-specific antigen
STI: Sexually transmitted infection
*T. vaginalis*: *Trichomonas vaginalis*
TvMIF: *Trichomonas vaginalis* Macrophage migration inhibitory factor
UTI: Urinary tract infection
