# Supplementary material for: Association between trichomoniasis and prostate and bladder diseases: a population-based case–control study
Source: Sci Rep. 2022 Sep 13;12:15358. doi: 10.1038/s41598-022-19561-2 (PMC9468537; doi:10.1038/s41598-022-19561-2)
Supplement: Supplementary file 1 — Supplementary Table S1. [file 41598_2022_19561_MOESM1_ESM.docx]

| **Table S1. Abbreviations and ICD-9-CM** | | |
| --- | --- | --- |
|  | **Abbreviation** | **ICD-9-CM** |
| **Study population:** BPH/prostate cancer, bladder cancer |  |  |
| BPH/prostate cancer |  |  |
| Benign prostatic hyperplasia | BPH | 600, 600.2, 600.9 |
| Prostate cancer |  | 185, 233.4, 236.5, V10.46 |
| Bladder cancer |  | 188, 188.0-188.5, 188.8-188.9, 233.7, 236.7, 239.4, V10.51 |
| **Exposure:** Trichomoniasis |  |  |
| Trichomonal vulvovaginitis |  | 131.01 |
| Trichomonal urethritis |  | 131.02 |
| Other urogenital trichomoniasis |  | 131.09 |
| Trichomoniasis of other specified sites |  | 131.8 |
| Trichomoniasis, unspecified |  | 131.9 |
| **Depression** |  | 296.2-296.3, 300.4, 311 |
| **Charlson comorbidity index revised** | CCI_R | CCI removed prostate cancer, bladder cancer |
